# Examining the Effects of a Brief, Fully Self-Guided Mindfulness Ecological Momentary Intervention on Empathy and Theory-of-Mind for Generalized Anxiety Disorder: Randomized Controlled Trial

**DOI:** 10.2196/54412

**Published:** 2024-05-24

**Authors:** Nur Hani Zainal, Michelle G Newman

**Affiliations:** 1 Department of Health Care Policy Harvard Medical School Boston, MA United States; 2 Department of Psychology National University of Singapore Singapore Singapore; 3 Department of Psychology The Pennsylvania State University University Park, PA United States

**Keywords:** empathy, theory-of-mind, mindfulness, ecological momentary intervention, generalized anxiety disorder, randomized controlled trial, mobile phone

## Abstract

**Background:**

The utility of brief mindfulness ecological momentary interventions (EMIs) to improve empathy and theory-of-mind has been underinvestigated, particularly in generalized anxiety disorder (GAD).

**Objective:**

In this randomized controlled trial, we aimed to examine the efficacy of a 14-day, fully self-guided, mindfulness EMI on the empathy and theory-of-mind domains for GAD.

**Methods:**

Adults (aged ≥18 y) diagnosed with GAD were randomized to a mindfulness EMI (68/110, 61.8%) or self-monitoring app (42/110, 38.2%) arm. They completed the Interpersonal Reactivity Index self-report empathy measure and theory-of-mind test (Bell-Lysaker Emotion Recognition Task) at prerandomization, postintervention, and 1-month follow-up (1MFU) time points. Hierarchical linear modeling was conducted with the intent-to-treat principle to determine prerandomization to postintervention (pre-post intervention) and prerandomization to 1MFU (pre-1MFU) changes, comparing the mindfulness EMI to self-monitoring.

**Results:**

Observed effects were generally stronger from pre-1MFU than from pre-post intervention time points. From pre-post intervention time points, the mindfulness EMI was more efficacious than the self-monitoring app on *fantasy* (the ability to imagine being in others’ shoes; between-intervention effect size: Cohen *d*=0.26, *P=*.007; within-intervention effect size: Cohen *d*=0.22, *P=*.02 for the mindfulness EMI and Cohen *d*=−0.16, *P=*.10 for the self-monitoring app). From pre-1MFU time points, the mindfulness EMI, but not the self-monitoring app, improved *theory-of-mind* (a window into others’ thoughts and intentions through abstract, propositional knowledge about their mental states, encompassing the ability to decipher social cues) and the *fantasy*, *personal distress* (stress when witnessing others’ negative experiences), and *perspective-taking* (understanding others’ perspective) empathy domains. The effect sizes were small to moderate (Cohen *d*=0.15-0.36; *P*<.001 to *P*=.01) for significant between-intervention effects from pre-1MFU time points. Furthermore, the within-intervention effect sizes for these significant outcomes were stronger for the mindfulness EMI (Cohen *d*=0.30-0.43; *P*<.001 to *P*=.03) than the self-monitoring app (Cohen *d*=−0.12 to 0.21; *P=*.001 to *P*>.99) from pre-1MFU time points. No between-intervention and within-intervention effects on *empathic concern* (feeling affection, compassion, and care when observing others in distress, primarily attending to their emotional well-being) were observed from pre-post intervention and pre-1MFU time points.

**Conclusions:**

The brief mindfulness EMI improved specific domains of empathy (eg, fantasy, personal distress, and perspective-taking) and theory-of-mind with small to moderate effect sizes in persons with GAD. Higher-intensity, self-guided or coach-facilitated, multicomponent mindfulness EMIs targeting the optimization of social relationships are likely necessary to improve the empathic concern domain in this population.

**Trial Registration:**

ClinicalTrials.gov NCT04846777; https://clinicaltrials.gov/study/NCT04846777

## Introduction

### Background

*Empathy* encompasses the capacity to comprehend and resonate with the emotional experiences of others, facilitating caregiving, knowledge sharing, and collaborative goal attainment [1,2]. Relatedly, *theory-of-mind* (ToM) offers insight into peoples’ thoughts and intentions via abstract, propositional knowledge concerning others’ mental states and encompasses the capacity to interpret social cues from others [3,4]. Both empathy and ToM play vital roles in comprehending the cognitive and emotional dynamics of others in social contexts [3]. Specifically, empathy and ToM have been linked to enhanced emotional well-being [5], stronger social connections [6], and improved social health [7]. Therefore, developing efficacious interventions to improve various empathy domains and ToM is essential.

In particular, it is possible that mindfulness-based interventions (MBIs) could be efficacious in enhancing empathy and ToM skills [8]. Broadly, numerous theorists asserted that practicing mindfulness diligently should inherently give rise to interpersonal growth, including kindness, compassion, and empathy [9]. Plausibly, the receptive, nonjudgmental attitude fostered by mindfulness exercises could be pivotal in nurturing empathy toward others [10,11]. In addition, practicing mindfulness offers a conducive environment for efforts to comprehend another individual while acknowledging the inherent incompleteness of such understanding [12]. Collectively, mindfulness practices via MBIs can enhance individuals’ attunement to others’ circumstances, deepen their emotional resonance, increase their willingness to engage with negative emotions, and foster compassion toward others’ experiences as an extension of self-compassion [13].

Supporting these theories, a narrative systematic review provided compelling evidence for the positive impact of MBIs on enhancing empathy in children and adolescents [14]. Similarly, in an open trial, an 8-week mindfulness-based cognitive therapy (MBCT) [15] among university students improved all empathy domains with moderate to large effects at pre-post intervention time points (Cohen *d*=0.48-1.19) [16]; however, the lack of a randomly assigned control intervention precluded the ability to make cause-effect inferences. Lending credence to this idea, meta-analytic data from experiments and randomized controlled trials (RCTs) indicated that MBIs were superior to control interventions in enhancing empathy in the healthy general population [17]. A particular case was how premedical and medical students’ participation in a mindfulness-based stress reduction (MBSR) [18] program significantly elevated their overall empathy levels compared to those in the control intervention among premedical and medical students [19]. Another study showed that a 3-month perspective-taking–focused MBI led to greater ToM performance compared to an emotion-focused MBI [20]. Similarly, a recent pilot trial suggested the promise of MBIs in enhancing ToM in individuals with psychotic disorders [21]. Overall, the literature alludes to the high likelihood of efficacy of MBIs in enhancing empathy and ToM domains for various populations.

However, most of these MBIs, such as the popular 8-week MBCT and MBSR programs, necessitated in-person weekly individual or group intervention sessions lasting 60 to 150 minutes with day-long, 6-hour meditation retreats [22]. Thus, evaluating the impact of brief MBIs is crucial in light of the growing popularity of concise MBIs, such as brief smartphone-delivered mindfulness ecological momentary interventions (EMIs) and web-based audio streams [23]. Scant yet positive evidence for the possibility of brief mindfulness EMIs’ efficacy on empathy-related outcomes exists across 2 studies. First, a 5-minute mindfulness induction enhanced both ToM and empathic concern among meditation-naive adults more than among control adults [24]. Second, an 11-minute MBI versus relaxation led to enhanced out-intervention altruism while adjusting for levels of in-intervention empathy in the US general population [25]. Despite that, to the best of our awareness, there is no evidence yet that these effects endured beyond these mindfulness inductions, emphasizing the need to assess the efficacy of brief mindfulness EMIs on longer-term outcomes.

In addition, a significant limitation in the current literature on the efficacy of MBIs on social-cognitive outcomes is the focus on healthy samples despite ample evidence demonstrating empathy and ToM problems in populations with psychiatric disorders, such as depression [26], eating disorders [27], and anxiety disorders [28]. In recent years, there has been growing recognition of dysregulation in social processes, particularly cognitive and emotional empathy, as significant transdiagnostic contributors to internalizing disorders such as major depressive disorder and generalized anxiety disorder (GAD) in terms of their etiology, diagnostic relevance, and maintenance [29,30]. A meta-analysis showed that increased anxiety symptoms (including excessive worry in GAD) correlated with reduced *perspective-taking* and heightened the ability to be deeply immersed in imaginative fictional worlds (or *fantasy*) [31]. In addition, higher anxiety symptoms were associated with less pronounced *empathic concern* for others' emotional well-being and others’ *personal distress*, the inclination to absorb or experience stress in reaction to others’ feelings. These relationships imply that suboptimal empathy and ToM processes could be a common factor across various forms of anxiety disorders. Nonetheless, basic science [31,32] and clinical research [33] attention on this topic has been more heavily weighted toward social anxiety disorder, obsessive-compulsive disorder [34], and posttraumatic stress disorder [35] than other anxiety disorders, such as GAD. This lacuna in the literature highlights the importance of investigating this topic in other anxiety disorders to inform novel treatment optimization efforts for GAD, a needed avenue that lacks exploration [36].

On that note, GAD, for which chronic excessive and uncontrollable worry features as a core symptom, is an understudied yet essential case in point. More specifically, individuals with GAD tended to have above-average ToM scores, but only for negative social cues when instructed to worry instead of relax [37]. In another study, those with GAD scored lower on a ToM assessment than non-GAD control intervention participants [38]. Furthermore, people with clinical depression and anxiety experienced increased worry symptoms on days when they pursued self-image goals above the sample average but encountered reduced worry symptoms when they pursued empathic, compassionate goals [39]. Broadly, persistent interpersonal challenges, including problems associated with empathy and social cognition, among a sizable subset of clients with GAD were linked to reduced progress both immediately after cognitive behavioral therapy (CBT) and during follow-up [40,41]. Altogether, these findings suggest that empathy and associated social-cognitive issues may be key maintenance factors in GAD. For these reasons, intensive psychotherapies integrating various theoretical modalities have been developed to enhance empathy in GAD [42], but their lengthiness and rigor preclude scalability. Collectively, suboptimal empathy, ToM, and interpersonal issues [42], coupled with a considerable reluctance to seek face-to-face mental health treatment in GAD [43], highlight the importance of determining whether brief mindfulness EMIs might be efficacious in targeting these social-cognitive outcomes for GAD.

This study was a secondary analysis of a published RCT, which showed that a brief mindfulness EMI reduced repetitive negative thinking and GAD severity and enhanced trait mindfulness and executive functioning among persons diagnosed with GAD [44]. Similar to this study, in prior research MBIs produced modest to moderate impacts on anxiety and depression symptoms [45], such as pathological worry [44,46-48]. In addition, a meta-analysis documented the substantial impact of MBIs on executive functioning in both nonclinical and clinical populations [49], such as persons with GAD [44]. Therefore, it is possible that a brief mindfulness EMI could help enhance ToM and empathy in those with GAD.

### Objective

We aimed to examine how a brief mindfulness EMI, compared to a self-monitoring app, might improve empathy and ToM in GAD. On the basis of the theories and evidence outlined, we hypothesized that a brief mindfulness EMI would be more efficacious than a self-monitoring app in improving 4 established empathy domains [50] and ToM [51] from pre-post time points. The empathy domains included *empathic concern* (feeling affection, compassion, and genuine care when observing others’ distress, with attentiveness centered on their emotional well-being); *fantasy* (the capacity for immersive engagement in fictional scenarios through imagination); *personal distress* (the anxiety and stress individuals experience when observing the adversity of others); and *perspective-taking* (understanding others’ vantage point) [52]. Moreover, we expected such improvements to occur in the longer term from prerandomization to 1-month follow-up (1MFU) time points.

## Methods

### Ethical Considerations

This study was approved by the Pennsylvania State University Institutional Review Board (approval STUDY00010664). Participants were compensated up to US $30, subject pool credit hours, or a mixture of both. We preregistered the RCT on ClinicalTrials.gov (NCT04846777) and the hypotheses of this study on the Open Science Framework [53]. This study was conducted in compliance with the American Psychological Association and Declaration of Helsinki ethical standards in treating human participants. Informed consent was obtained from participants as per the Penn State Institutional Review Board. All data were de-identified.

### Study Design

**Figure 1:**
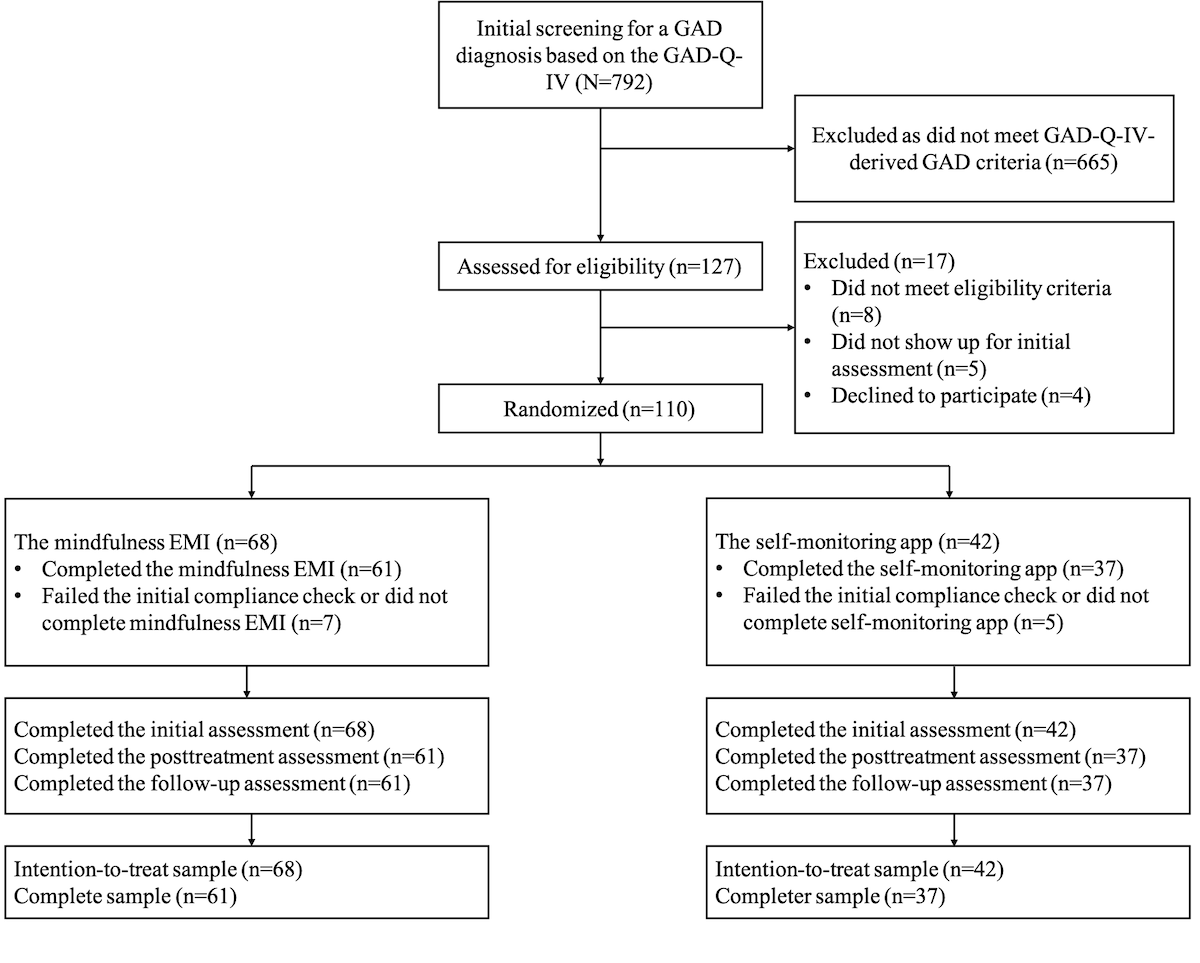
CONSORT (Consolidated Standards of Reporting Trials) flowchart of participant recruitment and progress. GAD: generalized anxiety disorder; GAD-Q-IV: Generalized Anxiety Disorder Questionnaire, Fourth Version; EMI: ecological momentary intervention.

We used a 2 (intervention: mindfulness EMI; control: self-monitoring app) × 3 (time: prerandomization, postintervention, and 1MFU) mixed methods design to assess the efficacy of the mindfulness EMI versus self-monitoring app on each distinct empathy and ToM outcome. Intervention was the between-subject factor, whereas time was the within-subject factor. We recruited 110 participants, with 61.8% (68/110) in the mindfulness EMI arm and 38.2% (42/110) in the self-monitoring app arm. Multimedia Appendix 1 [54-66] provides a comprehensive overview of the study’s methodology, including power analysis and reimbursement. Figure 1 displays the CONSORT (Consolidated Standards of Reporting Trials) diagram [67,68], illustrating participant enrollment and progression (refer to Multimedia Appendix 2 for the CONSORT-EHEALTH [Consolidated Standards of Reporting Trials of Electronic and Mobile Health Applications and Online Telehealth] checklist).

### Eligibility Criteria

Included participants were required to meet diagnostic criteria for GAD according to the *Diagnostic and Statistical Manual, Fifth Edition* (*DSM-5*) [54] and the GAD Questionnaire, Fourth Version (GAD-Q-IV) [55]. Initially, prospective participants underwent screening with the GAD-Q-IV. Subsequently, we invited those who scored at or above the clinical threshold for a brief clinical interview. The Anxiety and Related Disorders Interview Schedule (ADIS) for *DSM-5* [54,56] was used to establish their psychiatric diagnoses. Furthermore, participants were required to be aged at least 18 years, possess either an iPhone or Android phone, and provide informed consent. Exclusion criteria encompassed the presence of suicidal thoughts, manic episodes, disorders of psychosis, or substance use disorders.

### Participants

We recruited help-seeking participants diagnosed with GAD, who were not currently under the care of a mental health professional, from the local community and psychology subject pool. Table 1 displays the sociodemographic characteristics of the participants. Furthermore, the prevalence of comorbid diagnoses (eg, current or recurrent major depressive disorder, panic disorder, social anxiety disorder, obsessive compulsive disorder, posttraumatic stress disorder, alcohol use disorder, substance use disorder, anorexia nervosa, and binge-eating disorder) at baseline did not significantly differ between arms (all *P*>.05).

**Table 1:** Sociodemographic data of study participants in the mindfulness EMI^a^ and self-monitoring app arms (N=110).

Sociodemographic characteristics				Mindfulness EMI arm (n=68)		Self-monitoring app arm (n=42)		*P* value
**Continuous variables, mean (SD)**								
	Age (y)			20.53 (3.91)		21.24 (7.24)		.51
	14-item GAD-Q-IV^b^ score			9.52 (2.10)		9.94 (1.96)		.30
	**Treatment expectations**							
		Credibility	6.00 (1.39)		5.72 (1.58)		.34	
		Expectancy	43.46 (17.33)		44.29 (18.13)		.31	
**Categorical variables, n (%)**								
	**Gender orientation**							.85
		Women	10 (14.7)		5 (11.9)			
		Men	57 (83.8)		37 (88.1)			
		Declined to disclose	1 (1.5)		N/A^c^			
	**Race**							.99
		African American	5 (7.4)		1 (2.4)			
		Asian or Asian American	11 (16.2)		4 (9.5)			
		Declined to disclose	1 (1.5)		0 (0)			
		Hispanic	3 (4.4)		5 (11.9)			
		Other race	4 (5.9)		2 (4.8)			
		White	44 (64.7)		27 (64.3)			
	**Comorbid diagnoses**							
		Current major depressive episode	32 (47.1)		24 (57.1)		.30	
		Recurrent major depressive episode	25 (36.8)		20 (47.6)		.26	
		Current panic disorder	16 (23.5)		5 (11.9)		.13	
		Current social anxiety disorder	15 (22.1)		14 (33.3)		.19	
		Current OCD^d^	4 (5.9)		4 (9.5)		.48	
		Current PTSD^e^	9 (13.2)		4 (9.5)		.56	
		Current alcohol use disorder	7 (10.3)		1 (2.4)		.12	
		Current substance use disorder	3 (4.4)		1 (2.4)		.58	
		Current anorexia nervosa	0 (0)		0 (0)		N/A	
		Current binge-eating disorder	1 (1.5)		0 (0)		.39	

^a^EMI: ecological momentary intervention.

^b^GAD-Q-IV: Generalized Anxiety Disorder Questionnaire, Fourth Edition.

^c^N/A: not applicable.

^d^OCD: obsessive compulsive disorder.

^e^PTSD: posttraumatic stress disorder.

### Prerandomization Diagnostic Interview and Screening Assessment

#### Mental Disorder Diagnoses

The ADIS-5 [56] was used as a semistructured interview based on the *DSM-5* criteria [69]. Every ADIS-5 interview, whether face-to-face or via Zoom (Zoom Video Communications), was video-recorded and meticulously conducted by highly trained undergraduate and Bachelor of Arts–level assessors. All assessors had watched standardized training videos and completed quality assurance tests to maximize implementation fidelity to the study protocol. The study protocol was implemented remotely via Zoom during the COVID-19 pandemic. A subset comprising 40% (45/110) of these video recordings underwent a secondary evaluation by an independent, unbiased rater. We assessed the interrater reliability for GAD diagnoses. Any discrepancies were addressed through discussions and eventual consensus. The interrater agreement for GAD diagnosis was outstanding (Cohen κ=1.00), whereas, for other comorbid diagnoses and rule-outs, it ranged from very good to outstanding (average Cohen κ=0.75-0.98).

#### GAD Diagnosis

The screening for GAD used the 14-item GAD-Q-IV [55], which included a combination of dichotomous responses (“yes” or “no”) and continuous response formats (9-point Likert scales for items assessing the level of distress and interference attributed to GAD symptoms).

#### Intervention Arms

##### Mindfulness EMI App

During the first visit, assessors either exited the physical room (before the pandemic) or directed participants to turn off their Zoom audio and video before accessing the Qualtrics (Qualtrics International Inc) link to play the relevant intervention video (during the pandemic). In the mindfulness EMI, an instructional video featuring the principal investigator (a PhD-level clinical psychologist) was presented, delivering critical principles of evidence-based MBI protocols akin to the principles in MBSR [70]. Participants assigned to this condition were introduced to a precise conceptualization of mindfulness, with an explicit directive to immerse themselves entirely in their present circumstances and be engaged in activities. This segment was designed to endow individuals with chronic worry tendencies with proficiency in open monitoring, facilitating their capacity to attend to minute details. Following this, the video therapist taught techniques for deliberate, unhurried, rhythmic diaphragmatic breathing retraining, followed by a demonstration of its proper implementation. This element encompassed instruction in techniques for cultivating tranquility through controlled respiratory training and the cultivation of mindful qualities, such as observance without reactivity or judgment, drawing from the principles of MBCT [15]. Next, the video therapist underscored the significance and advantages of integrating mindfulness into daily routines. Subsequently, all assessors uniformly administered the 6-item Credibility and Expectancy Questionnaire [57]. Concluding this phase, assessors attentively addressed any inquiries about procedural but not the intervention aspects of the study (Multimedia Appendix 3). Participants were given a mindfulness EMI handout sent web-based in an automated manner via Qualtrics to preserve assessor blinding. The handout explicitly instructed them to review and practice its contents regularly.

The EMI encouraged individuals to engage in mindfulness exercises daily, precisely at 5 distinct intervals throughout the day: approximately 9 AM, noon, 3 PM, 6 PM, and 9 PM, over a span of 2 weeks. Within every instance of engagement with the mindfulness EMI, individuals participating in the program were provided with the following standard guidance instructions (Multimedia Appendices 1 and 3):

Pay attention to your breathing. Breathe in a slow, steady, and rhythmic manner. Stay focused on the sensations of the air coming into your lungs and then letting it out. As you are breathing, observe your experience as it is. Let go of judgments that do not serve you. Focus on the here and now. Attend to the small moments right now (e.g., reading a chapter, having a cool glass of water), as that is where enjoyment, peace, and serenity in life happen.

Individuals assessed their current levels of mindfulness (“To what extent are you experiencing the present moment fully?”), depression (“To what degree do you feel depressed right now?”), and anxiety (“To what degree do you feel keyed up or on edge right now?”) before and after receiving these instructions on a 9-point Likert scale, ranging from 1 (*not at all*) to 9 (*extremely*). Every mindfulness EMI notification concluded with the following motivating message to foster the enduring adoption of these skills:

Remember that the cultivation of mindfulness is lifelong. The goal of therapy is to be your own therapist. Practice mindfulness between the prompts and after you have completed this study.

##### Self-Monitoring App

In the self-monitoring app, the standardized video commenced with the principal investigator delineating self-monitoring as the heightened awareness of cognitive processes and emotional states. Subsequently, the video advanced by positing that the mere act of vigilantly tracking one’s thoughts and documenting any associated emotional distress could promote the cultivation of more adaptive cognitive patterns. Finally, the self-monitoring video conveyed the notion that the act of self-monitoring, in and of itself, possessed the potential to ameliorate feelings of anxiety. The foundational rationale for the self-monitoring condition was derived and adapted from the rationale used in a recent, brief app intervention [71]. This approach was crafted to closely mimic the mindfulness EMI protocol while excluding its hypothesized active, helpful components: acceptance, diaphragmatic breathing retraining, awareness toward subtle experiences, open monitoring, and sustained mindfulness practice. Consequently, the self-monitoring app deliberately abstained from any reference to the concept of mindfulness. This approach refrained from imparting explicit directives for participants to heighten their sensitivity and consciousness of their ongoing experiences; instead, its emphasis rested on vigilant monitoring of their thoughts and emotional responses. Moreover, participants were not tasked with focusing exclusively on their immediate activities because such a directive could inadvertently induce emotional state alterations. Although self-monitoring participants were directed to observe their cognitions and emotional states, there was a deliberate omission of instructions about accepting these thoughts and feelings as they manifested. Furthermore, the intervention did not include any guidance regarding breathing retraining techniques. It was not designed to elicit any sensations associated with relaxation typically linked to abdominal breathing. Participants were not instructed to engage in self-monitoring activities between the prescribed prompts or beyond the conclusion of the initial 2-week period. Consequently, self-monitoring, unlike mindfulness, was delimited to the active intervention period. The self-monitoring approach was also designed to control for credibility and expectancy effects, pre-empting regression to the mean and averting potential inflation of effect sizes that typically happen with a no-treatment or waitlist control (Multimedia Appendix 4).

In contrast to the more extensive mindfulness guidance provided by the mindfulness EMI, participants in the self-monitoring intervention received concise instructions 5 times a day (approximately 9 AM, noon, 3 PM, 6 PM, and 9 PM) over a span of 14 days (Multimedia Appendices 1 and 4): “Notice your thoughts and how distressing they may be.” We assessed individuals’ levels of mindfulness, depression, and anxiety using identical 9-point Likert scale questions both before and after each prompt in the self-monitoring sessions. Next, mirroring the mindfulness EMI, all assessors administered the 6-item Credibility and Expectancy Questionnaire. Furthermore, participants were provided a copy of the self-monitoring handout in an automated manner programmed via Qualtrics. Unlike the mindfulness EMI handout, this handout did not provide explicit instructions to review its contents routinely. In addition, the self-monitoring app was chosen as a placebo control as previous theory and research suggested that it would not be strong enough to elicit improvements in empathy and related social cognition relative to an MBI [72] yet could serve adequately as a placebo in an RCT [73].

### Prerandomization, Postintervention, and 1MFU Measures

#### Overview

Table 2 summarizes descriptive statistics of the empathy domains and ToM scores.

**Table 2:** Descriptive data of empathy and ToM^a^ variables across prerandomization, postintervention, and 1MFU^b^ time points in the mindfulness EMI^c^ (n=68) and self-monitoring (n=42) app arms (N=110).

		Values, mean (SE)^d^	Skewness	Kurtosis
**Mindfulness EMI at the prerandomization** **time point**				
	ToM (BLERT^e^)	17.85 (0.78)	−1.05	0.12
	Perspective taking (IRI^f^)	3.66 (0.28)	−0.22	−0.77
	Fantasy (IRI)	3.53 (0.29)	−0.27	−1.05
	Empathic concern (IRI)	3.97 (0.26)	−0.96	0.72
	Personal distress (IRI)	3.13 (0.27)	−0.16	−0.88
**S** **elf-monitoring** **at the prerandomization** **time point**				
	ToM (BLERT)	17.81 (0.34)	−0.07	−1.23
	Perspective taking (IRI)	3.62 (0.12)	−0.11	−1.25
	Fantasy (IRI)	3.71 (0.13)	−0.38	−0.04
	Empathic concern (IRI)	3.93 (0.12)	−0.35	−0.69
	Personal distress (IRI)	3.08 (0.12)	0.17	−0.79
**Mindfulness EMI at the postintervention** **time point**				
	ToM (BLERT)	17.94 (1.66)	−1.12	2.46
	Perspective taking (IRI)	3.68 (0.66)	−0.23	−0.94
	Fantasy (IRI)	3.87 (0.68)	−0.65	−0.83
	Empathic concern (IRI)	4.10 (0.60)	−1.31	1.71
	Personal distress (IRI)	3.21 (0.63)	−0.10	−1.10
**Self-monitoring at the postintervention** **time point**				
	ToM (BLERT)	17.95 (0.73)	−1.57	5.33
	Perspective taking (IRI)	3.60 (0.29)	0.14	−0.81
	Fantasy (IRI)	3.45 (0.30)	−0.57	−0.31
	Empathic concern (IRI)	3.74 (0.26)	−0.46	−0.41
	Personal distress (IRI)	2.80 (0.28)	0.01	−0.13
**Mindfulness EMI at the 1MFU** **time point**				
	ToM (BLERT)	19.13 (1.54)	−1.12	2.46
	Perspective taking (IRI)	3.90 (0.56)	−0.23	−0.94
	Fantasy (IRI)	3.85 (0.57)	−0.65	−0.83
	Empathic concern (IRI)	4.11 (0.51)	−1.31	1.71
	Personal distress (IRI)	3.38 (0.57)	−0.10	−1.10
**Self-monitoring at the 1MFU** **time point**				
	ToM (BLERT)	17.98 (0.68)	−0.04	−1.05
	Perspective taking (IRI)	3.62 (0.25)	−0.31	−0.77
	Fantasy (IRI)	3.57 (0.25)	−0.09	−0.43
	Empathic concern (IRI)	3.80 (0.22)	−0.57	−0.21
	Personal distress (IRI)	2.71 (0.25)	−0.11	−0.29

^a^ToM: theory-of-mind.

^b^1MFU: 1-month follow-up time point.

^c^EMI: ecological momentary intervention.

^d^These marginal means and SEs were derived from the hierarchical linear models.

^e^BLERT: Bell-Lysaker Emotion Recognition Task.

^f^IRI: Interpersonal Reactivity Index.

#### Empathy Domains

We used the 28-item Interpersonal Reactivity Index (IRI) [50] to assess 4 trait-level empathy domains, each with a subscale [74]. *Empathic concern* refers to experiencing affection and compassion when witnessing others’ distress, focusing on the well-being of others’ feelings (eg, “I am often quite touched by things that I see happen.”). *Fantasy* pertains to peoples’ imaginative ability to immerse themselves in fictional scenarios (eg, “I really get involved with the feelings of the characters in a novel.”). *Personal distress* refers to individuals’ feelings of anxiety and stress when witnessing others’ negative experiences (eg, “In emergency situations, I feel apprehensive and ill-at-ease.”). *Perspective-taking* entails comprehending others’ viewpoint (eg, “I sometimes try to understand my friends better by imagining how things look from their perspective.”). Each subscale comprised 4 items rated on a 5-point scale ranging from 1 (*never*) to 5 (*always*). Possible values of each subscale range from 1 to 5. The internal consistency (Cronbach α) of the IRI empathic concern scale was .85, .88, and .89 at prerandomization, postintervention, and 1MFU time points (fantasy: .82, .89, and .90; personal distress: .81, .90, and .89; and perspective tasking: .85, .88, and .89), respectively. Each IRI subscale has shown high convergent validity, acceptable discriminant validity [75], strong retest reliability, and good cross-cultural generalizability [76].

#### ToM Domains

We evaluated ToM using the Bell-Lysaker Emotion Recognition Task (BLERT) [51]. BLERT comprises 21 brief video clips where an actor enacts one of 3 dialogue options while expressing 7 distinct emotions (anger, disgust, fear, happiness, sadness, surprise, and no emotion). Participants selected the emotion that best corresponded to the emotion expressed by the actor from the 7 options displayed. An overall accuracy score was calculated (based on a score of 1 for each correct trial). Possible values range from 0 to 21. Cronbach α values of the BLERT total scale were .94, .89, and .95 at prerandomization, postintervention, and 1MFU time points, respectively. Furthermore, a prior review showed that the BLERT had high retest reliability, strong convergent validity, and good discriminant validity [77].

### Procedures

At visit 1, participants initially underwent the structured ADIS-5 interview. Afterward, eligible individuals completed a battery of prerandomization self-reports and behavioral and neuropsychological assessments in a manner that was counterbalanced, thus minimizing any potential for order-related biases. Assessors were uninformed regarding assigned arms, ensuring that the treatment assignment remained concealed from them throughout all study visits. They either left the physical room (before the COVID-19 pandemic) or provided clear instructions to participants to deactivate their Zoom audio and video before activating the Qualtrics link to play the pertinent treatment video (amid the COVID-19 pandemic). Participants installed the Personal Analytics Companion app, preloaded with either the mindfulness EMI or self-monitoring tool, onto their respective smartphones. The assessor was present if participants had questions about study procedures (eg, future study visits and technical questions about installing the app on their phone) but was not present when the participants were notified about their assigned intervention arm and its components. Participants were told that they would receive prompts at 5 designated times each day, at approximately 9 AM, noon, 3 PM, 6 PM, and 9 PM, over the ensuing 14 days, and these prompts could be flexibly adjusted based on their schedules. The prompts provided specific instructions to guide them to use mindfulness or self-monitoring strategies, contingent upon their assigned intervention. After 14 days, all participants reconvened at the laboratory (or on Zoom) for postintervention assessments and again during the 1MFU time point, during which they completed the requisite self-reports and neuropsychological battery. Participants received compensation in credit hours, monetary remuneration, or a combination of both (Multimedia Appendix 1). In addition, the research team performed a compliance verification on the seventh day and extended invitations to those participants who successfully passed this compliance assessment to continue in the treatment.

### Data Analyses

All analyses were based on the intent-to-treat principle. Of the 110 participants assigned to the mindfulness EMI (n=68, 61.8%) or self-monitoring app (n=42, 38.2%) arm, 98 (89.1%) participants conscientiously finished engaging with the 6-week study protocol, including all study visit assessments and ≥ 80% of the app prompts. A total of 12 (10.7%) out of 110 participants did not meet the seventh-day engagement threshold by failing to complete at least 80% of the EMI prompts from both arms over the 2-week intervention period. However, the data of these 12 (10.7%) participants were included in all analyses. The median number of prompts completed was 63 (range 0-70). To address missing values (29/1650, 1.76% of the entire data set), we conducted multiple imputation using chained equations with the predictive mean matching algorithm under the missing at-random assumption [78]. Specifically, we synthesized the data from 100 imputed data sets, each with a maximum of 10 iterations [79]. As another preprocessing step, we determined from a series of ordinary least square regressions that there were no significant between-intervention effects on BLERT ToM (β=.04, *P*=.92), IRI empathic concern (β=.05, *P*=.74), IRI fantasy (β=−.18, *P=*.26), IRI personal distress (β=.05, *P=*.74), and IRI perspective-taking (β=.04, *P=*.80) domains at prerandomization (baseline) time point. Furthermore, there were no significant differences in perceived credibility or expectancy between the interventions, with Cohen *d* values ranging from −0.05 to 0.19. Therefore, no covariates were included in the hierarchical linear modeling (HLM) predictive equations, given randomization success (Table 1).

HLM was used to compare interventions over time on all empathy-related outcomes. HLM considers the nesting of time points within participants, enabling the exploration of changes within and between participants over time (prerandomization, postintervention, and 1MFU time points) and by intervention (mindfulness EMI and self-monitoring app) [80,81]. Distinct models were used for each of the 5 outcomes. To assess efficacy of the mindfulness EMI versus self-monitoring app, we analyzed the time at level 1, delineating the first segment from pre-post intervention time points and a second segment from prerandomization to 1MFU (pre-1MFU) time points. The third segment from postintervention to 1MFU (post-1MFU) time points examined maintenance of any treatment gains. In each HLM model, fixed-effect predictors included intervention, time, and their interaction. The random-effect predictor was the intercept, which was coded for time (eg, prerandomization time point as “0” and postintervention time point as “1”), allowing for variability in average outcome values among participants. We used fitted models to calculate estimated mean scores at each time point. Cohen *d* effect sizes and their corresponding 95% CIs were computed to facilitate the interpretation of parameter estimates [82-84]. In this context, Cohen *d* values of 0.2, 0.5, and 0.8 represent small, moderate, and large effects, respectively.

## Results

### ToM: Propositional Knowledge of Others’ Emotional and Mental States

Despite nonsignificant intervention differences from pre-post intervention time points (Cohen *d*=−0.01, 95% CI −0.21 to 0.19; *P=*.91), the mindfulness EMI (vs the self-monitoring app) led to significantly greater change in ToM (BLERT scores) from pre-1MFU (Cohen *d*=0.25, 95% CI 0.05-0.45; *P=*.01; Table 3). From pre-post intervention, no significant changes in ToM occurred in either the mindfulness EMI (Cohen *d*=0.03, 95% CI −0.17 to 0.23; *P=*.75) or self-monitoring (Cohen *d*=0.03, 95% CI −0.16 to 0.23; *P=*.74) interventions. From pre-1MFU, the mindfulness EMI (Cohen *d*=0.43, 95% CI 0.23-0.63; *P*<.001) but not the self-monitoring app (Cohen *d*=0.06, 95% CI −0.14 to 0.25; *P=*.56) significantly enhanced ToM (Table 4). No substantial between-intervention and within-intervention effects on ToM emerged from post-1MFU time points (Tables S1 and S2 in Multimedia Appendix 5).

**Table 3:** Hierarchical linear models of between-intervention mindfulness ecological momentary intervention versus self-monitoring app effects on empathy and ToM^a^ variables from prerandomization to postintervention (pre-post intervention) and prerandomization to 1-month follow-up (pre-1MFU) time points.

			Pre-post intervention time points							Pre-1MFU time points				
			β^b^		*P* value		Cohen *d* (95% CI)		β			*P* value		Cohen *d* (95% CI)
**ToM (BLERT^c^)**														
	Intercept	17.81		<.001		4.94 (4.56 to 5.33)		17.81			<.001		4.94 (4.56 to 5.33)	
	Time	0.14		.71		0.04 (−0.16 to 0.23)		0.08			.62		0.05 (−0.15 to 0.24)	
	Intervention	0.04		.92		0.01 (−0.19 to 0.21)		0.04			.92		0.01 (−0.19 to 0.21)	
	Time × intervention	−0.05		.91		−0.01 (−0.21 to 0.19)		0.56			.01		0.25 (0.05 to 0.45)	
**Empathic concern (IRI^d^)**														
	Intercept	3.93		<.001		3.24 (2.95 to 3.53)		3.93			<.001		3.24 (2.95 to 3.53)	
	Time	−0.18		.23		−0.12 (−0.31 to 0.08)		−0.06			.25		−0.11 (−0.31 to 0.09)	
	Intervention	0.05		.74		0.03 (−0.16 to 0.23)		0.05			.74		0.03 (−0.16 to 0.23)	
	Time × intervention	0.30		.11		0.15 (−0.04 to 0.35)		0.13			.06		0.18 (−0.02 to 0.38)	
**Fantasy (IRI)**														
	Intercept	3.71		<.001		2.78 (2.51 to 3.05)		3.71			<.001		2.78 (2.51 to 3.05)	
	Time	−0.26		.13		−0.14 (−0.34 to 0.05)		−0.07			.25		−0.11 (−0.31 to 0.09)	
	Intervention	−0.18		.26		−0.11 (−0.3 to 0.09)		−0.18			.26		−0.11 (−0.3 to 0.09)	
	Time × intervention	0.60		.007		0.26 (0.06 to 0.46)		0.23			.004		0.28 (0.09 to 0.48)	
**Personal distress (IRI)**														
	Intercept	3.08		<.001		2.46 (2.20 to 2.71)		3.08			<.001		2.46 (2.20 to 2.71)	
	Time	−0.28		.08		−0.17 (−0.37 to 0.03)		−0.18			.005		−0.27 (−0.47 to −0.08)	
	Intervention	0.05		.74		0.03 (−0.16 to 0.23)		0.05			.74		0.03 (−0.16 to 0.23)	
	Time × intervention	0.36		.083		0.17 (−0.03 to 0.36)		0.31			<.001		0.36 (0.16 to 0.56)	
**Perspective taking (IRI)**														
	Intercept	3.62		<.001		2.77 (2.50 to 3.04)		3.62			<.001		2.77 (2.50 to 3.04)	
	Time	−0.02		.89		−0.01 (−0.21 to 0.18)		0.00			.99		0.00 (−0.20 to 0.20)	
	Intervention	0.04		.80		0.02 (−0.17 to 0.22)		0.04			.80		0.02 (−0.17 to 0.22)	
	Time × intervention	0.04		.84		0.02 (−0.18 to 0.22)		0.12			.13		0.15 (−0.05 to 0.34)	

^a^ToM: theory-of-mind.

^b^β: regression unstandardized parameter estimate.

^c^BLERT: Bell-Lysaker Emotion Recognition Task.

^d^IRI: Interpersonal Reactivity Index.

**Table 4:** Hierarchical linear models of within-intervention mindfulness EMI^a^ and self-monitoring app effects on empathy and ToM^b^ variables from prerandomization to postintervention (pre-post intervention) and prerandomization to 1-month follow-up (pre-1MFU) time points.

			Pre-post intervention time points							Pre-1MFU time points				
			β^c^		*P* value		Cohen *d* (95% CI)		β			*P* value		Cohen *d* (95% CI)
**ToM (BLERT^d^)**														
	Intercept (mindfulness EMI)	17.85		<.001		5.50 (5.08 to 5.92)		17.85			<.001		5.50 (5.08 to 5.92)	
	Time (mindfulness EMI)	0.09		.75		0.03 (−0.17 to 0.23)		0.64			<.001		0.43 (0.23 to 0.63)	
	Intercept (self-monitoring)	17.81		<.001		7.10 (6.58 to 7.62)		17.81			<.001		7.10 (6.58 to 7.62)	
	Time (self-monitoring)	0.14		.74		0.03 (−0.16 to 0.23)		0.08			.56		0.06 (−0.14 to 0.25)	
**Empathic concern (IRI^e^)**														
	Intercept (mindfulness EMI)	3.97		<.001		4.29 (3.94 to 4.64)		3.97			<.001		4.29 (3.94 to 4.64)	
	Time (mindfulness EMI)	0.12		.26		0.11 (−0.09 to 0.31)		0.07			.06		0.18 (−0.01 to 0.38)	
	Intercept (self-monitoring)	3.93		<.001		3.11 (2.82 to 3.40)		3.93			<.001		3.11 (2.82 to 3.40)	
	Time (self-monitoring)	−0.18		.28		−0.11 (−0.30 to 0.09)		−0.06			.35		−0.09 (−0.29 to 0.11)	
**Fantasy (IRI)**														
	Intercept (mindfulness EMI)	3.53		<.001		3.08 (2.80 to 3.37)		3.53			<.001		3.08 (2.80 to 3.37)	
	Time (mindfulness EMI)	0.34		.02		0.22 (0.03 to 0.42)		0.16			.002		0.30 (0.11 to 0.5)	
	Intercept (self-monitoring)	3.71		<.001		3.36 (3.06 to 3.66)		3.71			<.001		3.36 (3.06 to 3.66)	
	Time (self-monitoring)	−0.26		.10		−0.16 (−0.36 to 0.03)		−0.07			.22		−0.12 (−0.31 to 0.08)	
**Personal distress (IRI)**														
	Intercept (mindfulness EMI)	3.13		<.001		3.00 (2.72 to 3.28)		3.13			<.001		3.00 (2.72 to 3.28)	
	Time (mindfulness EMI)	0.07		.60		0.05 (−0.15 to 0.25)		0.12			.03		0.21 (0.02 to 0.41)	
	Intercept (self-monitoring)	3.08		<.001		2.75 (2.48 to 3.02)		3.08			<.001		2.75 (2.48 to 3.02)	
	Time (self-monitoring)	−0.28		.03		−0.21 (−0.41 to −0.02)		−0.18			.001		−0.33 (−0.53 to −0.13)	
**Perspective taking (IRI)**														
	Intercept (mindfulness EMI)	3.66		<.001		3.43 (3.13 to 3.74)		3.66			<.001		3.43 (3.13 to 3.74)	
	Time (mindfulness EMI)	0.02		.89		0.01 (−0.18 to 0.21)		0.12			.01		0.25 (0.06 to 0.45)	
	Intercept (self-monitoring)	3.62		<.001		2.95 (2.67 to 3.23)		3.62			<.001		2.95 (2.67 to 3.23)	
	Time (self-monitoring)	−0.02		.89		−0.01 (−0.21 to 0.18)		0.00			.99		0.00 (−0.20 to 0.20)	

^a^EMI: ecological momentary intervention.

^b^ToM: theory-of-mind.

^c^β: unstandardized regression parameter estimate.

^d^BLERT: Bell-Lysaker Emotion Recognition Task.

^e^IRI: Interpersonal Reactivity Index.

### Empathic Concern: Care About Others’ Psychological Well-Being

There was no significant difference between the mindfulness EMI and self-monitoring interventions in effects on empathic concern (IRI scores) from pre-post intervention (Cohen *d*=0.15, 95% CI −0.04 to 0.35; *P=*.11) and pre-1MFU (Cohen *d*=0.18, 95% CI −0.02 to 0.38; *P=*.06; Table 3) time points. From pre-post intervention, there were no significant changes in empathic concern in either the mindfulness EMI (Cohen *d*=0.11, 95% CI −0.09 to 0.31; *P=*.26) or self-monitoring (Cohen *d*=0.11, 95% CI −0.30 to 0.09; *P=*.28) interventions. Similarly, from pre-1MFU, no significant changes in empathic concern emerged in the mindfulness EMI (Cohen *d*=0.18, 95% CI −0.01 to 0.38; *P=*.06) or self-monitoring (Cohen *d*=−0.09, 95% CI −0.29 to 0.11; *P=*.35; Table 4) interventions. No significant between-intervention and within-intervention effects on empathic concern emerged from post-1MFU time points (Tables S1 and S2 in Multimedia Appendix 5).

### Fantasy: the Ability to Imagine Others’ Experiences

The mindfulness EMI (vs self-monitoring app) led to greater effects on fantasy (IRI scores) from pre-post intervention (Cohen *d*=0.26, 95% CI 0.06-0.46; *P=*.007) and pre-1MFU (Cohen *d*=0.28, 95% CI 0.09-0.48; *P=*.004; Table 3). From pre-post intervention, the mindfulness EMI (Cohen *d*=0.22, 95% CI 0.03-0.42; *P=*.02) but not the self-monitoring app (Cohen *d*=−0.16, 95% CI −0.36 to 0.03; *P=*.10) generated significant improvement in fantasy. Similarly, from pre-1MFU, the mindfulness EMI (Cohen *d*=0.30, 95% CI 0.11-0.50; *P=*.002) but not the self-monitoring app (Cohen *d*=−0.12, 95% CI −0.31 to 0.08; *P=*.22) significantly enhanced fantasy (Table 4). Maintenance of gains occurred from post-1MFU time points (Tables S1 and S2 in Multimedia Appendix 5).

### Personal Distress: Feeling Distress When Observing Others' Adverse Experiences

There were no significant intervention differences In personal distress (IRI scores) from pre-post intervention (Cohen *d*=0.17, 95% CI −0.03 to 0.36; *P=*.08). At the same time, from pre-post intervention, the self-monitoring app (Cohen *d*=−0.21, 95% CI −0.41 to −0.02; *P=*.03) but not the mindfulness EMI (Cohen *d*=0.05, 95% CI −0.15 to 0.25; *P=*.60) significantly reduced personal distress. There were significant differences between mindfulness EMI and self-monitoring interventions from pre-1MFU time points (Cohen *d*=0.36, 95% CI 0.16-0.56; *P*<.001; Table 3). Although the mindfulness EMI significantly increased personal distress (Cohen *d*=0.21, 95% CI 0.02-0.41; *P=*.03), the self-monitoring app significantly decreased it (Cohen *d*=−0.33, 95% CI −0.53 to −0.13; *P=*.001; Table 4). No significant between-intervention and within-intervention effects on personal distress emerged from post-1MFU time points (Tables S1 and S2 in Multimedia Appendix 5).

### Perspective Taking: Comprehending Others’ Viewpoint

There were no significant differences between mindfulness EMI and self-monitoring on perspective-taking (IRI scores) from pre-post intervention (Cohen *d*=0.02, 95% CI −0.18 to 0.22; *P=*.84) and pre-1MFU (Cohen *d*=0.15, 95% CI −0.05 to 0.34; *P=*.13; Table 3). From pre-post intervention, no significant changes in perspective-taking emerged in the mindfulness EMI (Cohen *d*=0.01, 95% CI −0.18 to 0.21; *P=*.89) and self-monitoring (Cohen *d*=−0.01, 95% CI −0.21 to 0.18; *P=*.89) interventions. However, from pre-1MFU, the mindfulness EMI (Cohen *d*=0.25, 95% CI 0.06-0.45; *P=*.01) but not the self-monitoring app (Cohen *d*=0.00, 95% CI −0.20 to 0.20; *P=*.99; Table 4) yielded significant improvements in perspective-taking. No significant between-intervention and within-intervention effects on perspective taking emerged from post-1MFU time points (Tables S1 and S2 in Multimedia Appendix 5).

## Discussion

### Principal Findings and Comparison With Prior Work

Partially supporting our hypothesis, brief self-guided mindfulness EMI displayed longer-term efficacy from pre-1MFU time points compared to self-monitoring in improving 3 of the 5 examined social-cognitive domains: ToM, the capacity for imaginative immersion in various scenarios (*fantasy*), and experiencing distress when observing others’ adverse situations (*personal distress*). Moreover, the mindfulness EMI, but not self-monitoring, enhanced perspective-taking from pre-1MFU time points despite the lack of between-intervention effects. An unexpected outcome was that neither of the interventions yielded effects on empathic concern. Our findings cannot be attributed to sociodemographic and comorbid psychiatric diagnoses, as those variables did not differ between compared conditions at prerandomization. On the whole, these outcomes suggest *specificity*, instead of globality, in the impact of brief mindfulness EMI and self-monitoring on ToM and trait-level empathy domains in the context of GAD. Potential theories are put forth to advance clinical science on this underinvestigated yet essential topic.

Why did brief mindfulness EMI but not self-monitoring enhance ToM (the ability to interpret social cues and represent abstract propositional knowledge about others’ emotional and mental states), the capacity to envision being in another person’s shoes (*fantasy*), and perspective-taking across 6 weeks? These outcomes extended reports of positive correlations between mindfulness and the ability to understand the emotions of self and others cognitively (cognitive empathy) [85-87]. The facets of nonjudgmental contemplation and a focus on the present moment emphasized by the mindfulness EMI could wield significant influence in cultivating empathic responses. Such mindfulness EMI instructions might augment cognitive capabilities, such as adopting another’s perspective [9] and fostering a pragmatic understanding of human distress [88]. Collectively, our findings and these testable interpretations aligned harmoniously with previous 8- to 12-week MBI RCTs [89,90], highlighting the benefits of the mindfulness EMI’s brevity on these social-cognitive domains.

Moreover, although the brief mindfulness EMI improved negative affective empathy (*distress*) or anxiety and stress experienced when confronted with the negative experiences of others, self-monitoring worsened it from pre-1MFU time points. This finding was inconsistent with a study showing that a brief mindfulness EMI was associated with increased *cognitive empathy* but decreased *affective empathy* (the capacity to share in the emotional experiences of others vicariously) [91]. Perhaps the brief mindfulness EMI evaluated in this study might have safeguarded against desensitization to suffering or developing callousness, enabling one to witness others’ adverse experiences without feeling overwhelmed [92]. The opposite pattern occurred in the self-monitoring intervention, probably due to instructions to focus inwardly on one’s stressful feelings and thoughts without asking the participant to have regard for others’ difficulties.

Contrary to expectations, the brief mindfulness EMI did not outperform the self-monitoring app in improving empathic concern (experiencing affection and compassion when witnessing others’ distress). Furthermore, no changes in empathic concern occurred from pre-post intervention and pre-1MFU time points. Similarly, a prior experiment found that a 5-minute mindfulness induction, compared to 2 control conditions, did not yield any discernible impact on empathic responses [93]. On the basis of prior RCTs that tested empirically-supported 8-week MBIs [94,95], a tenable account is a need for lengthier, higher-intensity (fully self-guided or coach-guided), multicomponent mindfulness EMIs focused on interpersonal relationships to evoke measurable improvement in empathic concern [96,97]. Future studies could test this conjecture and evaluate dose-response relationships between mindfulness EMI duration or intensity and its impact on various empathy domains. Alternatively, as prior research showed that people diagnosed with GAD experienced notably above-average levels of empathic concern [98], perhaps other empathy domains apart from empathic concern were more malleable to change via mindfulness EMIs in this population. A future RCT that recruited participants with both GAD and below-average levels of empathic concern would provide a direct test of this hypothesis.

As far as we know, there has been no other study that examined the effects of MBI on empathy in individuals with GAD. One prior study found that CBT but not MBI led to changes in positive affective empathy in individuals with social anxiety disorder compared to a waitlist control condition [33]. Furthermore, a change in positive affective empathy was a mediator of change in CBT but not in MBSR [33]. Given that we found changes in empathy and ToM domains relative to a control condition, perhaps enhancements in ToM and empathy domains are mechanisms of change of the effects of MBIs on GAD symptom alteration and related outcomes but not on social anxiety disorder. Conducting a mediation analysis to determine if changes in empathy and ToM domains were potential mechanisms of how brief mindfulness EMIs might improve GAD symptoms was beyond the scope of this study. However, a future report using the same data set will aim to explore this possibility.

### Limitations and Strengths

Interpreting the findings within the context of the limitations herein is imperative for an in-depth understanding. First, it is worth noting that the 2-week intervention duration might be inadequate to elicit immediate enhancements in empathy domains; nevertheless, the results showed greater promise for habitual worriers from pre-1MFU time points. Second, we did not incorporate assessments to gauge mindfulness EMI participants’ ongoing mindfulness skill use during the post-1MFU evaluation. Future investigations should, therefore, test the potential impact of continuous mindfulness practices, even in the absence of recurring guidance via the mindfulness EMI, on any discernible between-intervention effects during follow-up assessments. Third, we only assessed ToM and 4 empathy domains; extensive theories have suggested that MBIs are helpful in increasing other social-cognitive outcomes, such as altruism and prosocial behaviors [99], and these theories should be explored in GAD and related disorders. On that note, clinical science can profit from examining the effect of mindfulness EMIs on multimodal measures of empathy, such as proinflammatory cytokines, oxytocin, and neuroimaging indexes [100,101]. Finally, recruiting more diverse samples is required to establish cross-cultural generalizability [102,103].

Nonetheless, this study demonstrated notable strengths, including the gold standard RCT design featuring an active control intervention, a commendably high compliance rate, the recruitment of a clinically diagnosed sample with ample statistical power, and the incorporation of comprehensive assessments. Furthermore, it is noteworthy that our attrition rate, standing at a mere 11%, fell below the typical 24% to 50% range observed in RCTs delivered through smartphones [104,105]. Our relatively minimal attrition may be attributed to the prorated reimbursement schedule design.

### Conclusions

In summary, these findings suggest that brief self-guided mindfulness EMIs such as ours hold promise as scalable solutions to enhance specific empathy domains (*perspective-taking* and *fantasy* or the ability to immerse oneself imaginatively in diverse situations experienced by others), *personal distress* (feeling distressed when witnessing unfavorable circumstances affecting others), and *ToM* (the skill to decipher social signals) for persons with GAD. Nonetheless, mindfulness EMI did not elicit between-intervention and within-intervention effects on *empathic concern* (experiencing warmth, compassion, and sincere concern while observing another person’s distress) for this population. To advance our understanding of the genuine therapeutic efficacy of mindfulness EMIs (brief or prolonged) on social-cognitive domains, it becomes crucial to discern the specific subinterventions for whom it benefits, the contexts in which it exerts its effects, and when it may be contraindicated or inadvisable. Consequently, these efforts might address global challenges to social integration, anxiety disorders, and other psychological well-being aspects encountered by communities.

## Appendix

Multimedia Appendix 1 Study methodology details.

Multimedia Appendix 2 CONSORT E-HEALTH checklist.

Multimedia Appendix 3 Screenshots of the intervention arm.

Multimedia Appendix 4 Screenshots of the control arm.

Multimedia Appendix 5 Postintervention to 1-month follow-up outcomes.

## Abbreviations

1MFU: 1-month follow-up
ADIS: Anxiety and Related Disorders Interview Schedule
BLERT: Bell-Lysaker Emotion Recognition Task
CBT: cognitive behavioral therapy
CONSORT: Consolidated Standards of Reporting Trials
CONSORT-EHEALTH: Consolidated Standards of Reporting Trials of Electronic and Mobile Health Applications and Online Telehealth
DSM-5: Diagnostic and Statistical Manual, Fifth Edition
EMI: ecological momentary intervention
GAD: generalized anxiety disorder
GAD-Q-IV: Generalized Anxiety Disorder Questionnaire, Fourth Version
HLM: hierarchical linear modeling
IRI: Interpersonal Reactivity Index
MBCT: mindfulness-based cognitive therapy
MBI: mindfulness-based intervention
MBSR: mindfulness-based stress reduction
RCT: randomized controlled trial
ToM: theory-of-mind
