# Supplementary material for: Examining the Effects of a Brief, Fully Self-Guided Mindfulness Ecological Momentary Intervention on Empathy and Theory-of-Mind for Generalized Anxiety Disorder: Randomized Controlled Trial
Source: JMIR Ment Health. 2024 May 24;11:e54412. doi: 10.2196/54412 (PMC11161716; doi:10.2196/54412)

**Multimedia Appendix 3**

# Screenshots of Intervention Arm

## Screenshots for mindfulness ecological momentary intervention (MEMI) arm


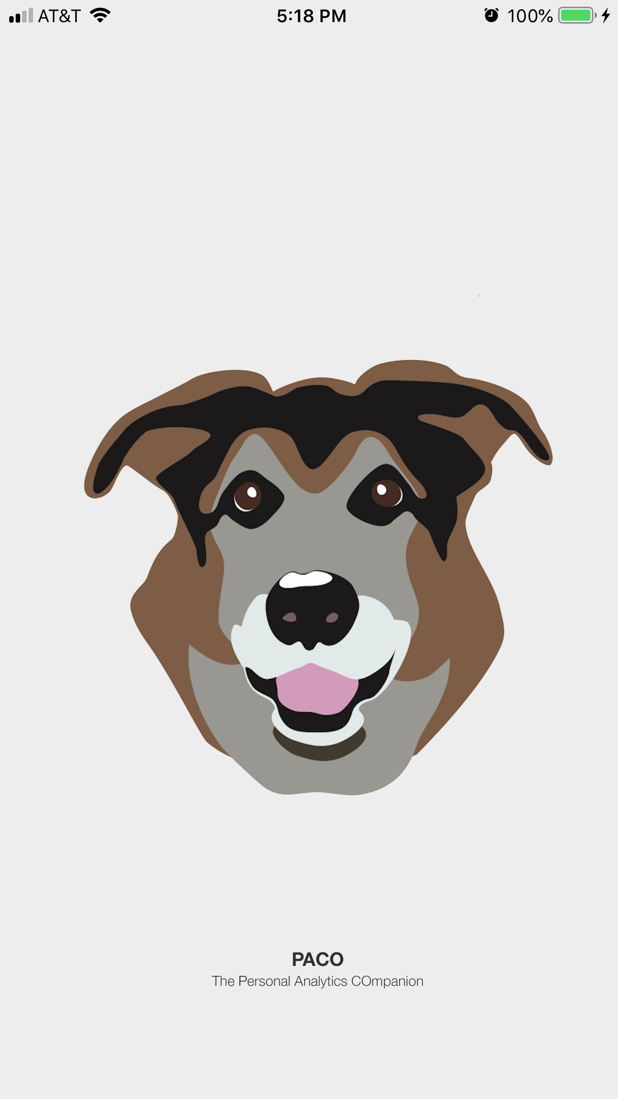

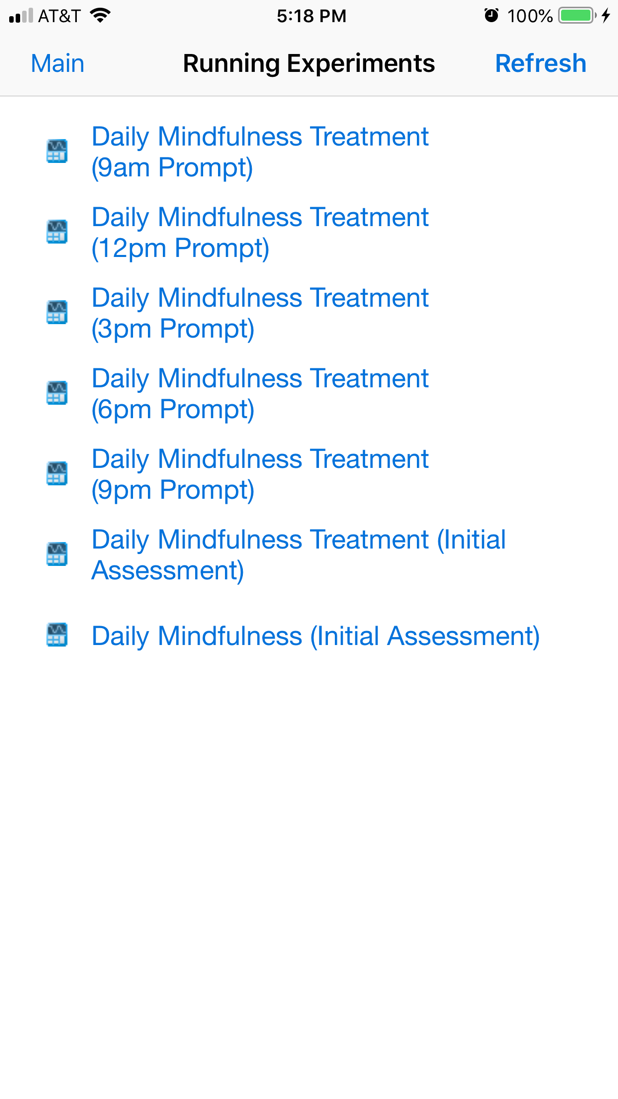


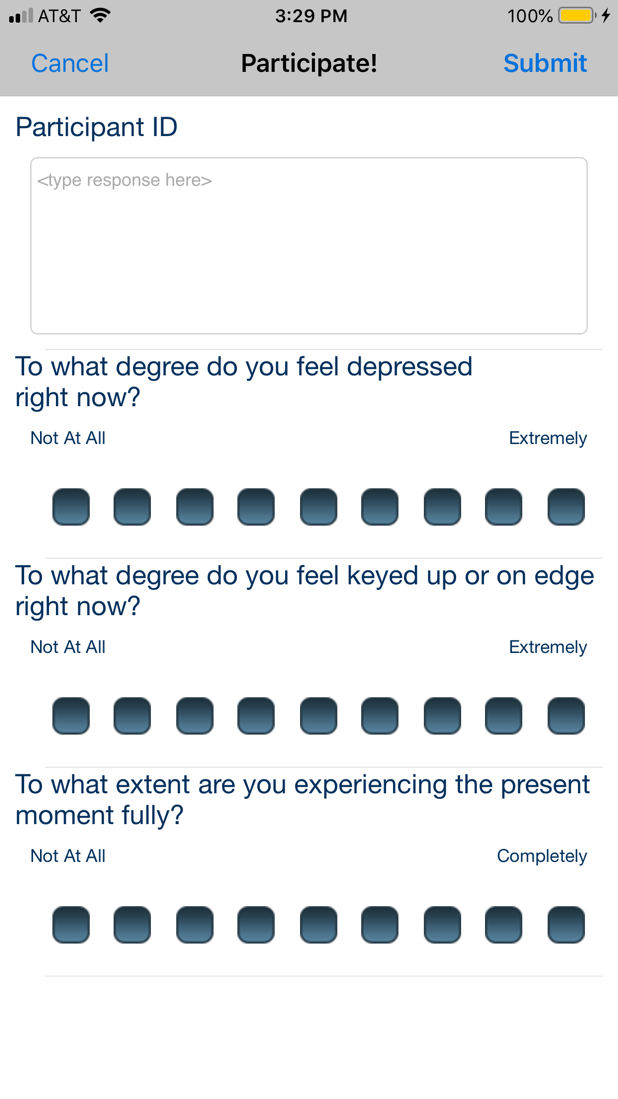

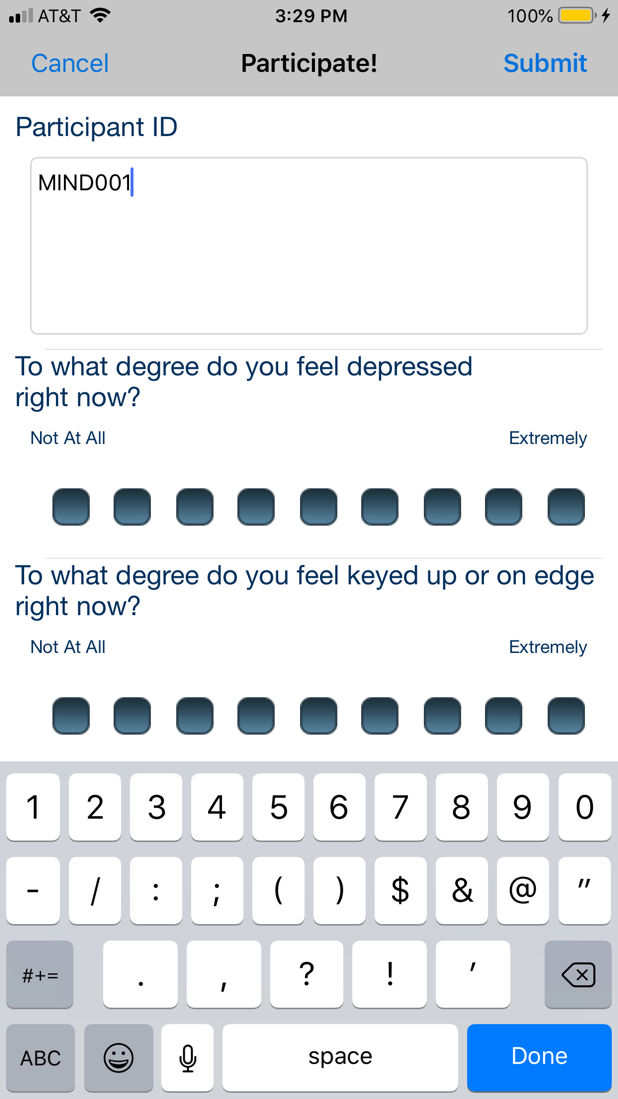


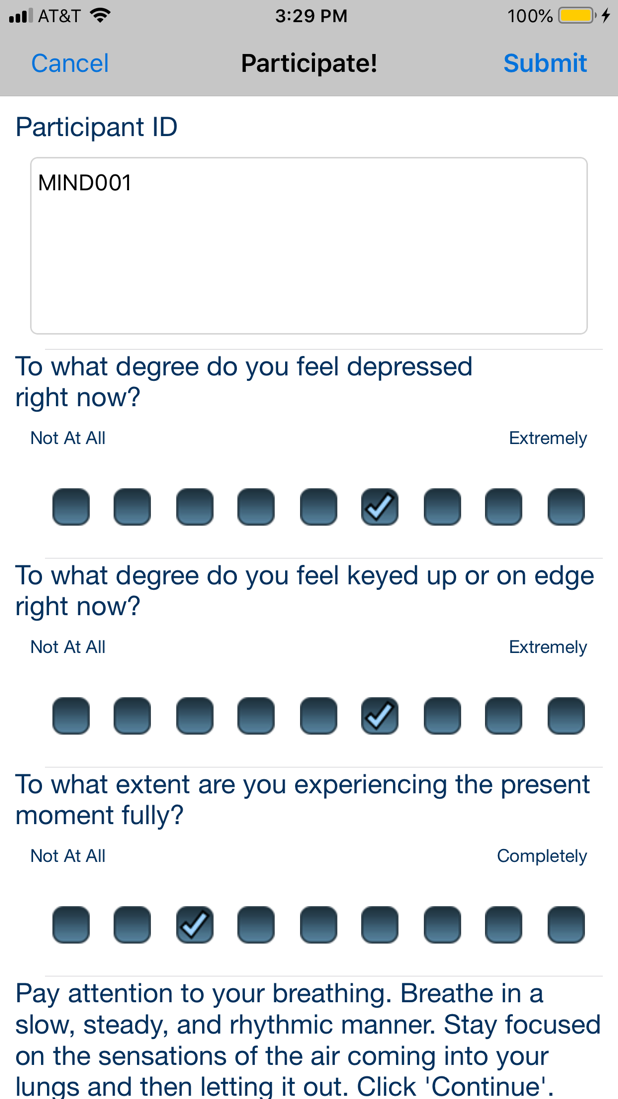

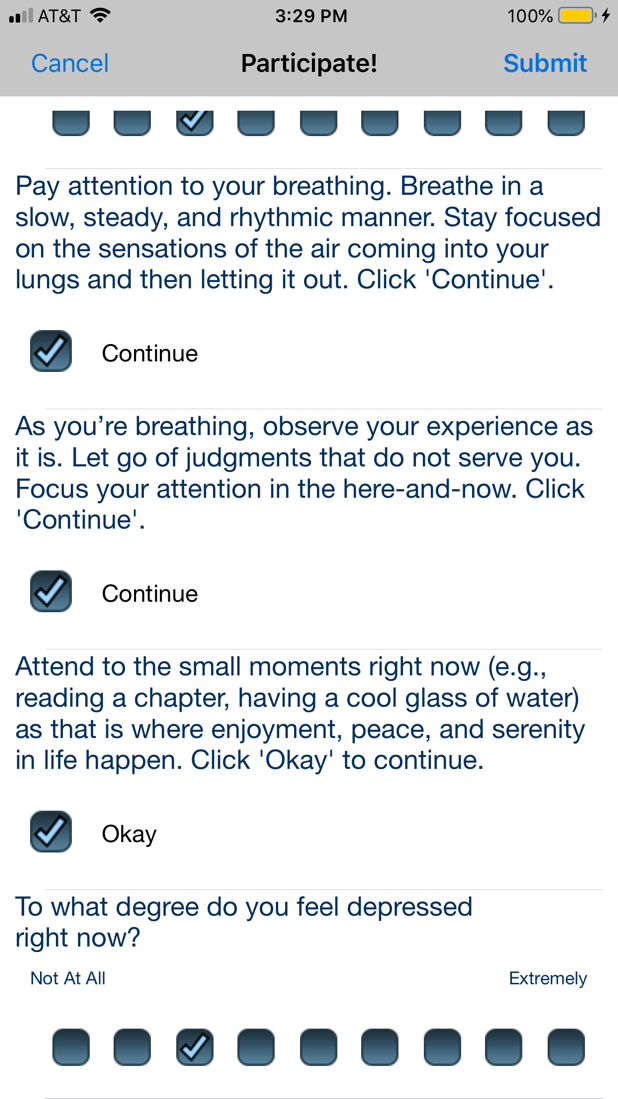


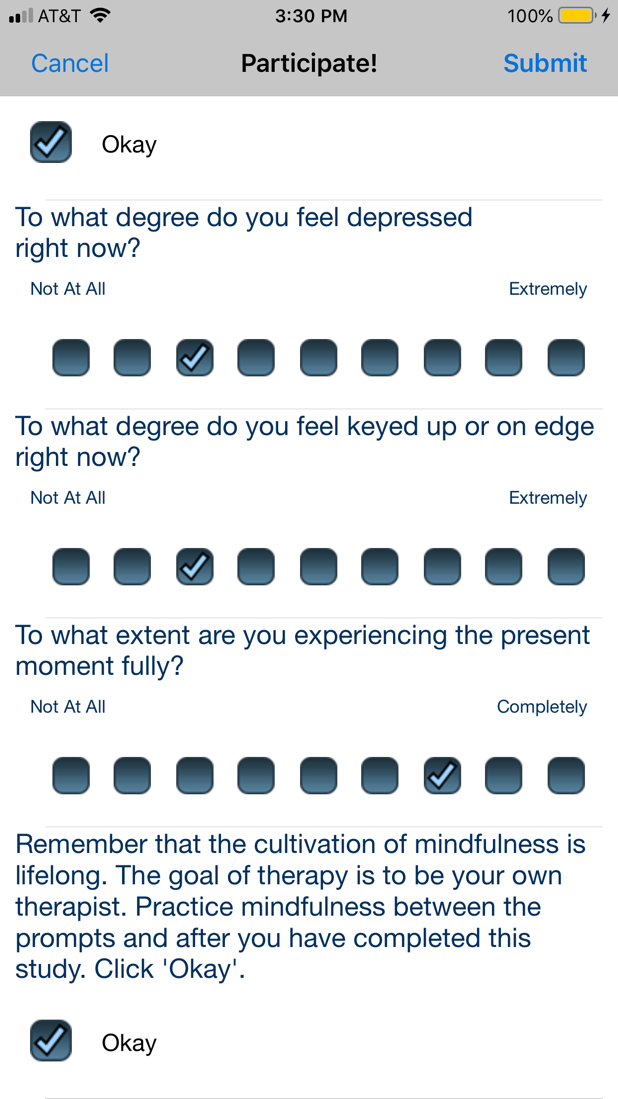

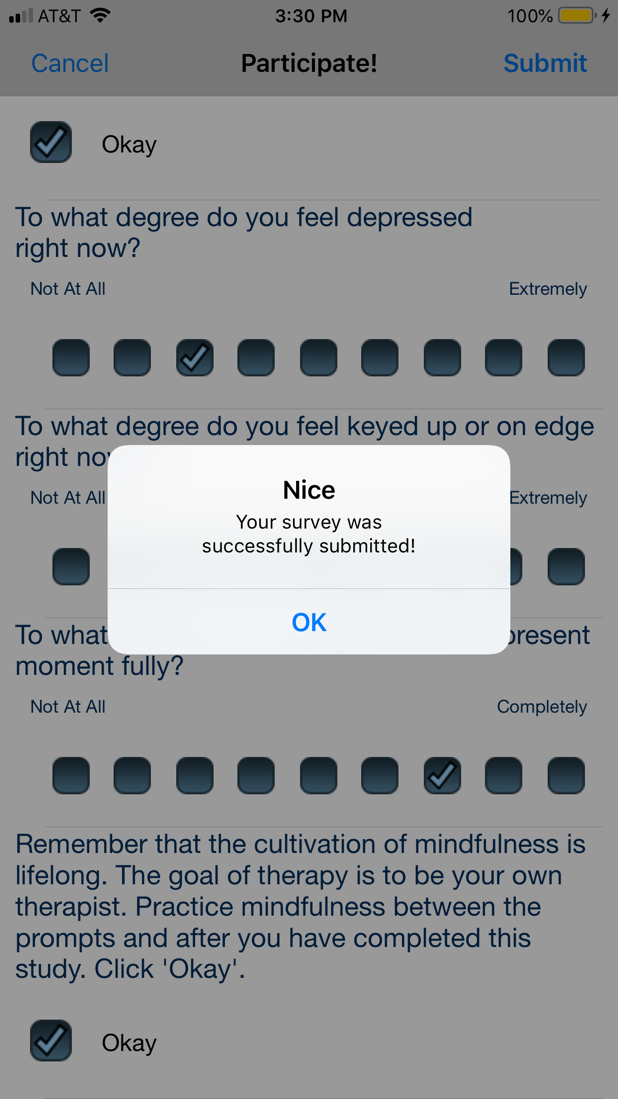

Supplement: Multimedia Appendix 3 [file mental_v11i1e54412_app3.docx]
