# Supplementary material for: Examining the Effects of a Brief, Fully Self-Guided Mindfulness Ecological Momentary Intervention on Empathy and Theory-of-Mind for Generalized Anxiety Disorder: Randomized Controlled Trial
Source: JMIR Ment Health. 2024 May 24;11:e54412. doi: 10.2196/54412 (PMC11161716; doi:10.2196/54412)
Supplement: Multimedia Appendix 5 [file mental_v11i1e54412_app5.docx]

**Multimedia Appendix 5**

**Post-1-Month Follow-Up Outcomes**

Table S1

*Hierarchical linear models of between-group mindfulness ecological momentary intervention (MEMI) vs. self-monitoring app (SM) effects on empathy and theory-of-mind (ToM) variables from post-intervention to 1-month follow-up (1MFU)*

|  | Post-intervention-1MFU | | | | | |
| --- | --- | --- | --- | --- | --- | --- |
|  | β | (*SE*) | *df* | *P* | *d* | [95% CI] |
| 1. *ToM (Bell-Lysaker Emotion Recognition Task; BLERT)* | | | | | | |
| Intercept | 17.93 | (0.68) | 108 | .000 | 2.50 | [2.24, 2.76] |
| Time | 0.02 | (0.37) | 108 | .948 | 0.01 | [-0.19, 0.20] |
| Group | -1.18 | (0.87) | 108 | .178 | -0.13 | [-0.33, 0.07] |
| Time x Group | 1.17 | (0.47) | 108 | .014 | 0.24 | [0.04, 0.44] |
| 1. *Empathic concern (Interpersonal Reactivity Index; IRI)* | | | | | | |
| Intercept | 3.69 | (0.24) | 108 | .000 | 1.13 | [0.98, 1.29] |
| Time | 0.06 | (0.15) | 108 | .704 | 0.03 | [-0.12, 0.17] |
| Group | 0.40 | (0.30) | 108 | .188 | 0.10 | [-0.05, 0.24] |
| Time x Group | -0.04 | (0.19) | 108 | .817 | -0.02 | [-0.16, 0.13] |
| 1. *Fantasy (Interpersonal Reactivity Index; IRI)* | | | | | | |
| Intercept | 3.33 | (0.31) | 108 | .000 | 0.77 | [0.62, 0.92] |
| Time | 0.12 | (0.20) | 108 | .542 | 0.04 | [-0.10, 0.19] |
| Group | 0.56 | (0.40) | 108 | .165 | 0.10 | [-0.04, 0.25] |
| Time x Group | -0.14 | (0.26) | 108 | .582 | -0.04 | [-0.18, 0.10] |
| 1. *Personal distress (Interpersonal Reactivity Index; IRI)* | | | | | | |
| Intercept | 2.89 | (0.28) | 108 | .000 | 0.73 | [0.59, 0.88] |
| Time | -0.09 | (0.18) | 108 | .626 | -0.04 | [-0.18, 0.11] |
| Group | 0.15 | (0.36) | 108 | .689 | 0.03 | [-0.12, 0.17] |
| Time x Group | 0.26 | (0.23) | 108 | .254 | 0.08 | [-0.06, 0.23] |
| 1. *Perspective-taking (Interpersonal Reactivity Index; IRI)* | | | | | | |
| Intercept | 3.57 | (0.27) | 108 | .000 | 0.96 | [0.81, 1.11] |
| Time | 0.02 | (0.17) | 108 | .884 | 0.01 | [-0.13, 0.16] |
| Group | -0.11 | (0.34) | 108 | .738 | -0.02 | [-0.17, 0.12] |
| Time x Group | 0.20 | (0.21) | 108 | .356 | 0.07 | [-0.08, 0.21] |

β, regression unstandardized parameter estimate; *SE*, standard error of β; *df*, model degrees of freedom; *P*, *p*-value; *d*, Cohen's *d* effect sizes; CI, confidence interval of *d*.

Table S2

*Hierarchical linear models of within-group mindfulness ecological momentary intervention (MEMI) and self-monitoring app (SM) effects on empathy and theory-of-mind (ToM) variables from post-intervention to 1-month follow-up (1MFU)*

|  | Post-intervention-1MFU | | | | | |
| --- | --- | --- | --- | --- | --- | --- |
|  | β | (*SE*) | *df* | *P* | *d* | [95% CI] |
| 1. *ToM (Bell-Lysaker Emotion Recognition Task; BLERT)* | | | | | | |
| Intercept (MEMI) | 16.75 | (0.51) | 67 | .000 | 3.11 | [2.82, 3.40] |
| Time (MEMI) | 1.19 | (0.29) | 67 | .000 | 0.40 | [0.20, 0.60] |
| Intercept (SM) | 17.93 | (0.73) | 41 | .000 | 2.33 | [2.08, 2.58] |
| Time (SM) | 0.02 | (0.37) | 41 | .949 | 0.01 | [-0.19, 0.2] |
| 1. *Empathic concern (Interpersonal Reactivity Index; IRI)* | | | | | | |
| Intercept (MEMI) | 4.09 | (0.18) | 67 | .000 | 2.17 | [1.93, 2.42] |
| Time (MEMI) | 0.01 | (0.11) | 67 | .913 | 0.01 | [-0.19, 0.21] |
| Intercept (SM) | 3.69 | (0.25) | 41 | .000 | 1.42 | [1.20, 1.64] |
| Time (SM) | 0.06 | (0.15) | 41 | .706 | 0.04 | [-0.16, 0.23] |
| 1. Fantasy (Interpersonal Reactivity Index; IRI) | | | | | | |
| Intercept (MEMI) | 3.88 | (0.25) | 67 | .000 | 1.46 | [1.24, 1.68] |
| Time (MEMI) | -0.02 | (0.16) | 67 | .912 | -0.01 | [-0.21, 0.19] |
| Intercept (SM) | 3.33 | (0.30) | 41 | .000 | 1.06 | [0.85, 1.27] |
| Time (SM) | 0.12 | (0.19) | 41 | .512 | 0.06 | [-0.13, 0.26] |
| 1. *Personal distress (Interpersonal Reactivity Index; IRI)* | | | | | | |
| Intercept (MEMI) | 3.03 | (0.25) | 67 | .000 | 1.14 | [0.93, 1.35] |
| Time (MEMI) | 0.17 | (0.16) | 67 | .277 | 0.10 | [-0.09, 0.3] |
| Intercept (SM) | 2.89 | (0.21) | 41 | .000 | 1.32 | [1.10, 1.54] |
| Time (SM) | -0.09 | (0.13) | 41 | .514 | -0.06 | [-0.26, 0.13] |
| 1. *Perspective-taking (Interpersonal Reactivity Index; IRI)* | | | | | | |
| Intercept (MEMI) | 3.46 | (0.22) | 67 | .000 | 1.53 | [1.30, 1.75] |
| Time (MEMI) | 0.22 | (0.13) | 67 | .102 | 0.16 | [-0.04, 0.35] |
| Intercept (SM) | 3.57 | (0.26) | 41 | .000 | 1.31 | [1.09, 1.52] |
| Time (SM) | 0.02 | (0.16) | 41 | .881 | 0.01 | [-0.18, 0.21] |

β, regression unstandardized parameter estimate; *SE*, standard error of β; *df*, model degrees of freedom; *P*, *p*-value; *d*, Cohen's *d* effect sizes; CI, confidence interval of *d*.
